# Total hip arthroplasty using a three-dimensional porous titanium acetabular cup: an examination of micromotion using subject-specific finite element analysis

**DOI:** 10.1186/s12891-021-04174-z

**Published:** 2021-03-26

**Authors:** Takaki Miyagawa, Kazu Matsumoto, Shingo Komura, Haruhiko Akiyama

**Affiliations:** Department of Orthopaedic Surgery, Gifu University School of Medicine, Yanagido 1-1, Gifu, 501-1194 Japan

**Keywords:** Finite element analysis, Total hip arthroplasty, Three-dimensional porous structure, Radiolucent line

## Abstract

**Background:**

We investigated the mid-term clinical and radiological results of total hip arthroplasty (THA) using a three-dimensional (3D) porous titanium cup and analyzed the micromotion at the interface of the cup using subject-specific finite element (FE) analysis.

**Methods:**

We evaluated 73 hips of 65 patients (6 men and 59 women; mean age at the time of surgery, 62.2 years; range, 45–86 years) who had undergone THA using a 3D porous titanium cup. Clinical evaluations were performed using the Japanese Orthopaedic Association (JOA) hip score system. We assessed the fixation of the acetabular component based on the presence of radiolucent lines and cup migration using anteroposterior radiographs. Subject-specific FE models were constructed from computed tomography data.

**Results:**

The JOA score improved from a preoperative mean of 52.2 (range, 23–82) to a mean of 87.8 (range, 71–100) at the final follow-up. None of the patients underwent revisions during the follow-up period. Radiolucent lines were observed in 26 cases (35.6%) and frequently appeared at DeLee and Charnley Zone 3. Following the FE analysis, the micromotion at DeLee and Charnley Zone 3 was significantly larger than that at Zone 2. Furthermore, micromotion was large in the groups in which radiolucent lines appeared at Zone 3.

**Conclusions:**

The mid-term clinical outcome of THA using a 3D porous titanium cup was excellent. However, radiolucent lines frequently appeared at DeLee and Charnley Zone 3. FE analysis indicated that micromotion was large at the same site, strongly suggesting that it contributes to the emergence of radiolucent lines. The 3D porous titanium cups are useful in THA, and with improvements focused on micromotion, we anticipate better long-term outcomes.

## Background

According to national registration reports, the most common reason for revision surgery after total hip arthroplasty (THA) is aseptic loosening [1–3]. Similarly, the primary reason for revision surgery in Japan is aseptic loosening of the acetabular component [4]. Loosening of the acetabular component is caused by osteolysis due to the abrasion powder of the polyethylene liner or poor fixation. To obtain long-term stable acetabular component fixation, biological fixation is required, and therefore, the initial fixation force and bone infiltration into the component porous region, are important [5, 6].

Since 2000, acetabular components with a three-dimensional (3D) structure in the porous region have been developed, and their use is associated with experimental efficacy and good survival rates [7–12]. The features of the 3D porous structure include a high friction coefficient and high porosity. Scratch fit caused by a high friction coefficient suppresses micromotion [5, 13, 14] and higher porosity promotes bone infiltration into the interior of the porous region, resulting in long-term stability due to the interlocking of the acetabular component and bone [15].

However, in clinical reports of implants with a 3D porous structure, a high rate of radiolucent lines was observed [16–18]. Radiolucent lines are said to appear because of the fibrous fixation between the bone and components and occur with micromotion of ≥150 μm [19].

The purposes of this study were to investigate the mid-term clinical results of THA using a 3D porous component, including the presence or absence of radiolucent lines, and to analyze the micromotion at the interface of the 3D acetabular component using finite element (FE) analysis.

## Methods

**Fig. 1:**
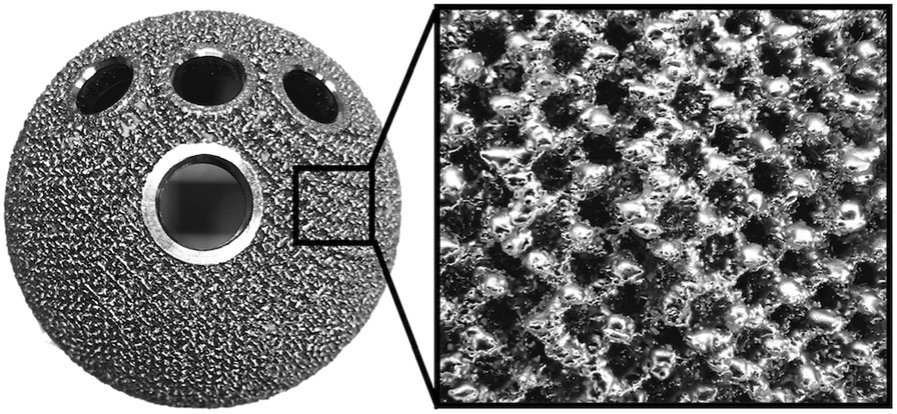
A porous, hexagonal, tridimensional, multiplane structure obtained using an electron beam melting technique

Between May 2014 and May 2016, 66 patients (74 hips) underwent primary THA using the SQRUM TT (Fig. 1). One patient (one hip) was lost to follow-up. We, therefore, evaluated 73 hips of 65 patients (6 men and 59 women). Causative hip diseases for THA included dysplastic osteoarthritis of 63 hips, idiopathic osteonecrosis of 2 hips, rapidly destructive coxarthropathy of 2 hips, post-traumatic arthritis of 2 hips, rheumatoid arthritis of 1 hip, and post-infective arthritis of 1 hip. All patients underwent pre- and postoperative computed tomography (CT). Preoperative CT was performed at the preoperative examination, and postoperative CT was performed 6 weeks after surgery. The implant used was the SQRUM TT (SQRUM TT™ socket; Kyocera, Kyoto, Japan). Cement or cementless stems were effectively used, depending on the shape and properties of the femur. SQRUM TT was only used when Kyocera’s femoral stem and a cementless cup could be implanted. The specific design element of this prosthesis was a hexagonal tridimensional multiplane structure, which was made from titanium alloy (Ti6Al4V), obtained using an electron beam melting technique. The porosity was 60%, the pore size was 640 μm, and the depth of porous structure was 1.2 mm (Fig. 1). Because of the design, the prosthesis had a solid cellular structure that was highly porous, mimicking natural cancellous bone and related elasticity (1.12 Gpa) [20, 21].

The operations were performed in a laminar-flow operating room, and patients received 1.0 g of cephalosporin intravenously immediately before surgery for antibiotic prophylaxis. Surgical procedures were performed using a direct lateral approach with the patient in the lateral position [22–24]. Acetabular reaming was performed using the same diameter as the outer diameter of the installation cup, and three screws were used to reinforce the initial fixing force.

### Clinical evaluation

Clinical evaluations were performed preoperatively and at the final follow-up using the Japanese Orthopaedic Association hip score system, in which the maximum score of 100 points was divided into a ‘pain’ score (40 points), ‘range of motion’ score (20 points), ‘walking ability’ score (20 points), and ‘activity of daily living’ score (20 points). A higher score indicated a better condition [25, 26]. Intraoperative and postoperative complications were also investigated.

### Radiological evaluation

We assessed the fixation of the acetabular component based on the presence of radiolucent lines and cup migration using anteroposterior radiographs. The presence of radiolucent lines in the three acetabular zones of DeLee and Charnley [27] was evaluated using three types divided into five stages as advocated by Long et al. [28] Cup migration was investigated by changing the position of the acetabular component and the inclination angle [29]. The superior position of the cup center was also measured in relation to the distance between the bottom of the teardrop and the center of the cup. The lateral position of the cup center was also measured as the horizontal distance between the bottom of the teardrop and the perpendicular point on the inter-teardrop line from the center of the cup. Cup inclination was determined using a horizontal reference line drawn through the base of the teardrops [30].

### FE analysis

**Fig. 2:**
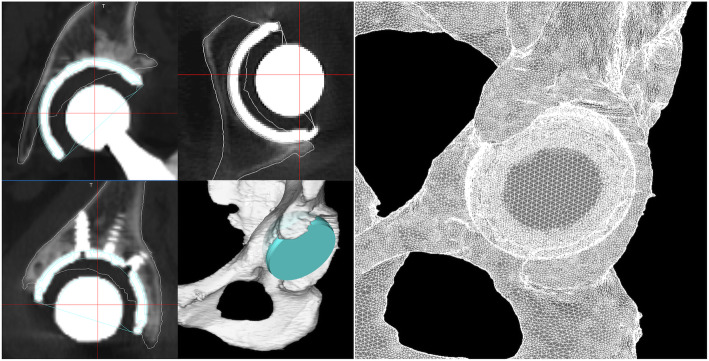
Finite element model of a pelvis and acetabular component created to match the postoperative location. By creating a pelvic region of interest from preoperative computed tomography (CT) and synthesizing it with implant Computer Aided Design data on postoperative CT, the postoperative condition is reproduced without the effect of halation caused by the implant

CT scans (Brilliance64; Philips, Best, Netherlands) of the pelvis were acquired pre- and postoperatively. The scanner settings were approximately 120 kV, 0.68 × 0.68 × 1-mm voxel size. FE models of the pelvis were generated from pre- and postoperative CT data using Mechanical Finder (MF) version 11.0 (Research Centre of Computational Mechanics, Inc., Tokyo, Japan). Only region of interest (ROI) models were semi-automatically constructed in 3D-planning software; Zed View version 12.0 (LEXI, Tokyo, Japan). The acetabular component Computer Aided Design data were precisely placed in the pelvic ROI model by matching the postoperative CT perfectly in Zed View. A composite ROI of the pelvis and acetabular components was imported into the MF for analysis (Fig. 2).

This created an FE model that indicated the shape of the individual bone and the distribution of its density [31]. Within this software, FE mesh models were generated using Ansys ICEM CFD version 19.0 (Ansys Inc., Canonsburg, Pennsylvania), and modelled as a 0.5–2.0 mm tetrahedral element for cancellous and medial cortical bone.

The analysis was performed with the elastic modulus of the porous region of the SQRUM TT being 1.12 Gpa and Poisson’s ratio being 0.44. When the SQRUM TT was implanted, the tremor under load was calculated for each region. The pelvic FE model consisted of approximately 2.5 million elements. The elastic modulus of the bone was determined from the CT density values using the equation proposed by Keyak et al. [32] (Table 1). The bone Poisson’s ratio was 0.40. The model was analyzed assuming an interface where the pelvic and acetabular components are in contact with a coefficient of friction of 1.02. Regarding the applied load condition, assuming one-leg standing, the upper region of the pelvis was completely fixed, and 1980 N, which is 3.3-fold the body weight based on Pauwels’ theory [33], was added to the functional axis from the knee joint center to the femoral head center.

**Table 1:** Equations used to calculate the elastic modulus of the bone

Bone density	Elastic modulus (MPa)
*P* = 0	0.001
0 < *p* ≤ 0.27	33900p^2.20^
0.27 < *p* < 0.6	5307 p + 469
0.6 ≤ *p*	10200p^2.01^

The elastic modulus of the bone is based on computed tomography density values, using the equations proposed by Keyak et al. [32]

p (g/cm^3^) = (H. U. + 1.4246) × 0.001/1.058 (H. U. > − 1) = 0.0 (H. U. ≤ − 1)

*H. U.* Hounsfield units.

Eight FE models were prepared using 50-mm cups and 28-mm heads, four of which had radiolucent lines clinically. The elemental nodes of the porous part included in the radius of 3 mm were extracted at the three points corresponding to the zones of DeLee and Charnley, and the relative movement distance with the elemental nodes of the adjacent bones corresponding to these was defined as micromotion.

### Statistical analysis

Statistical analyses were conducted using GraphPad Prism Version 5.01 (GraphPad Software, La Jolla, CA). Intraclass correlation coefficients (ICCs) were used to assess the intra- and inter-observer (two observers) reliabilities of the cup inclination and the cup migration measurements. ICC values can range from 0 to 1, with a higher value indicating better reliability. ICC values were analyzed in accordance with a previously described semi-quantitative scale (0–0.20, slight agreement; 0.21–0.40, fair; 0.41–0.60, moderate; 0.61–0.80, substantial; and 0.81–1.0, almost perfect) [34]. A paired t-test was used to analyze changes in radiological measurements. The micromotion of the three groups was compared using one-way analysis of variance and the Dunn-Bonferroni test. An unpaired t-test was performed to compare the micromotions of the groups with and without the radiolucent line. A *p*-value of < 0.05 indicated statistical significance. Additionally, statistical power analysis was performed using G∗Power 3.1.9.4 with an α level of 5%.

## Results

### Demographic characteristics

Patient demographics are shown in Table 2. Briefly, the mean age of the patients at the time of surgery was 62.2 years (range, 45–86 years), and the mean clinical follow-up was 5.3 years (range, 4.3–6.3 years).

**Table 2:** Patient demographics

Sex (n)	
Male	6 (7 hips)
Female	59 (66 hips)
Age, mean (range) (years)	62.2 (45–86)
Height, mean (range) (cm)	152.9 (133.8–171.6)
Weight, mean (range) (kg)	54.4 (34.0–73.8)
Body mass index, mean (range) (kg/m^2^)	23.3 (15.1–30.8)
Crowe classification (n)	
Group I	59
Group II	10
Group III	3
Group IV	1

### Clinical results

Paired t-tests showed that the Japanese Orthopaedic Association hip score significantly improved from a preoperative mean of 52.2 (range, 23–82) to a mean of 87.8 (range, 71–100) at the final follow-up (*p* < 0.01). One patient developed sciatic nerve disorder, but the symptoms improved during follow-up. Mild ectopic ossification and dislocation were each observed in one patient. No patients underwent revisions during the follow-up period.

### Radiological results

The mean (standard deviation [SD], range) cup inclination angles were 44.4° (4.75, 33.1–57.3) immediately after the operation and 44.8° (4.95, 33.1–59.4) at the final observation. There was a significant difference between the angle immediately after surgery and at the final observation (*p* < 0.05). There was a case in which the angle increased by ≥3°, but no migration was observed. There was no significant change in the movement distance of the center of the cup in the vertical (mean, 0.16 mm, *p* = 0.123) and horizontal (mean, 0.17 mm, *p* = 0.27) directions from immediately after surgery until the final observation.

**Fig. 3:**
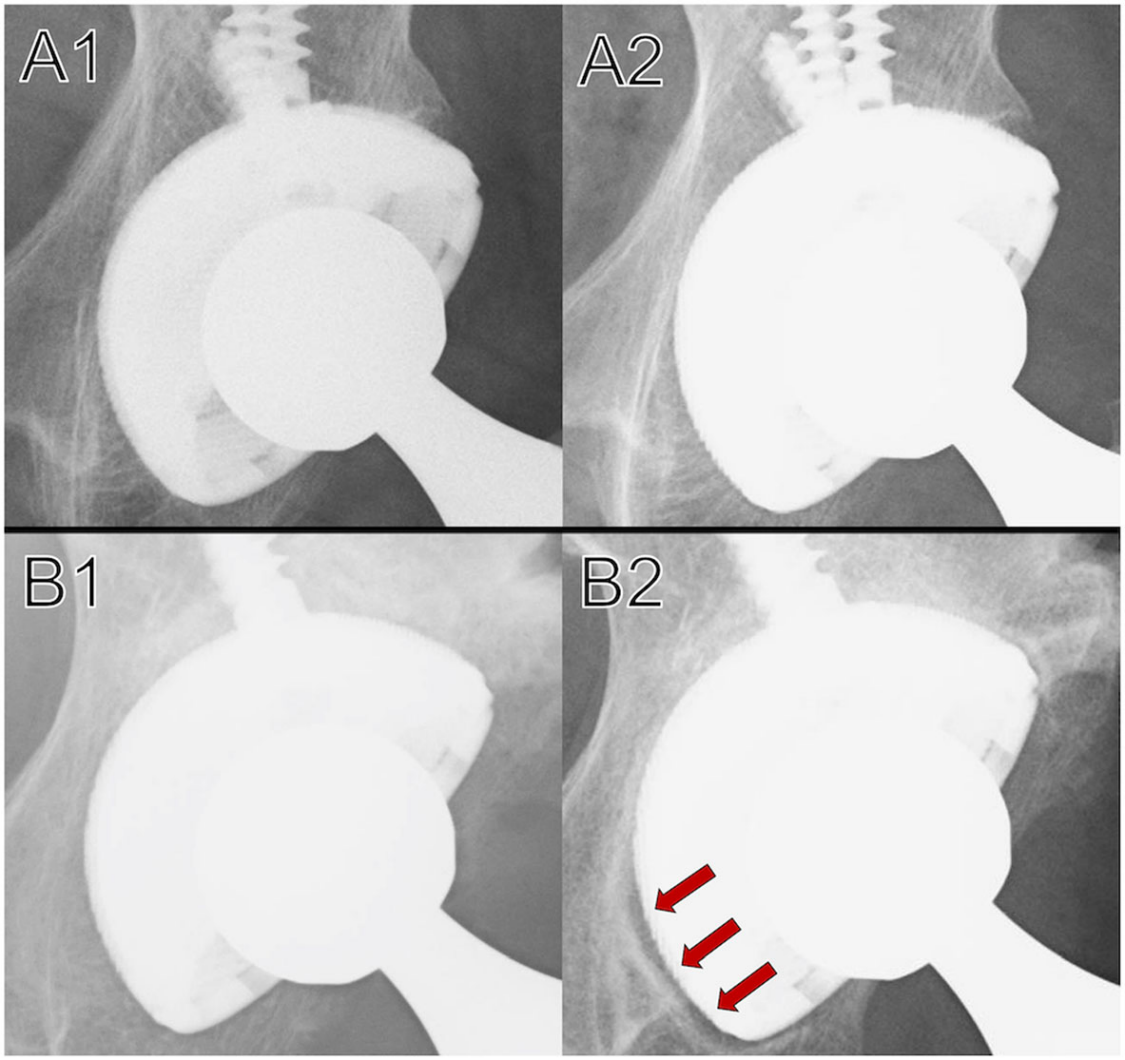
Radiograph images in which radiolucent lines did not appear and where they did appear. A1, B1: There is no initial gap on the anteroposterior radiograph immediately after surgery. A2: There is no radiolucent line around the cup 2 years after surgery. B2: Two years after the operation, radiolucent lines are observed in DeLee and Charnley Zone 3 (red arrows)

Initial gaps were observed in 14 hips, but gap filling occurred in all cases during follow-up. Radiolucent lines were observed in only one DeLee and Charnley zone in 21 hips, in two zones in four hips, and in three zones in one hip (Fig. 3). No enlargement of the radiolucent line was observed by the final observation. When comparing the zones, the radiolucent lines appeared most frequently in Zone 3 (Tables 3, 4).

**Table 3:** Modified DeLee and Charnley skeletal fixation score for the acetabulum

	Fixation grade	Radiolucency by zone	Hips (n)
Bone ingrowth, stable	IA	None	47
	IB	One zone	21
	IC	Two zone	4
Fibrous ingrowth, stable	II	Complete RLL < 2 mm all zones	1
Fibrous fixation, unstable	III	Progressive RLL Zone 3, complete RLL	0
		≥2 mm all zones, or socket migration	

*RLL* Radiolucent line

**Table 4:** Total number and area of radiolucent lines

DeLee and Charnley Zone	n
Zone 1	6
Zone 2	4
Zone 3	22

Two intra-observer ICCs were calculated; both were ≥ 0.9 for the radiographic measurements. The inter-observer ICCs were ≥ 0.8. These values indicate an agreement between different radiographic measurements.

### FE analysis

**Fig. 4:**
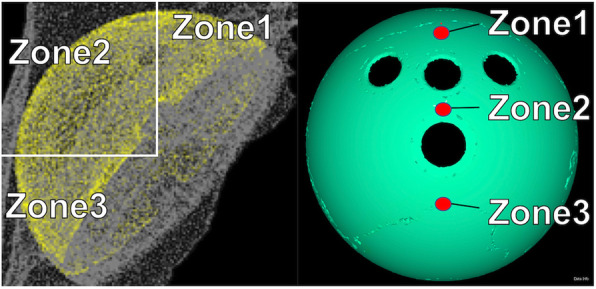
Finite element model of the porous part used in micromotion analysis. Element nodes were extracted from the locations corresponding to DeLee and Charnley Zones 1 to 3 of the porous region, and micromotion analysis was performed

**Fig. 5:**
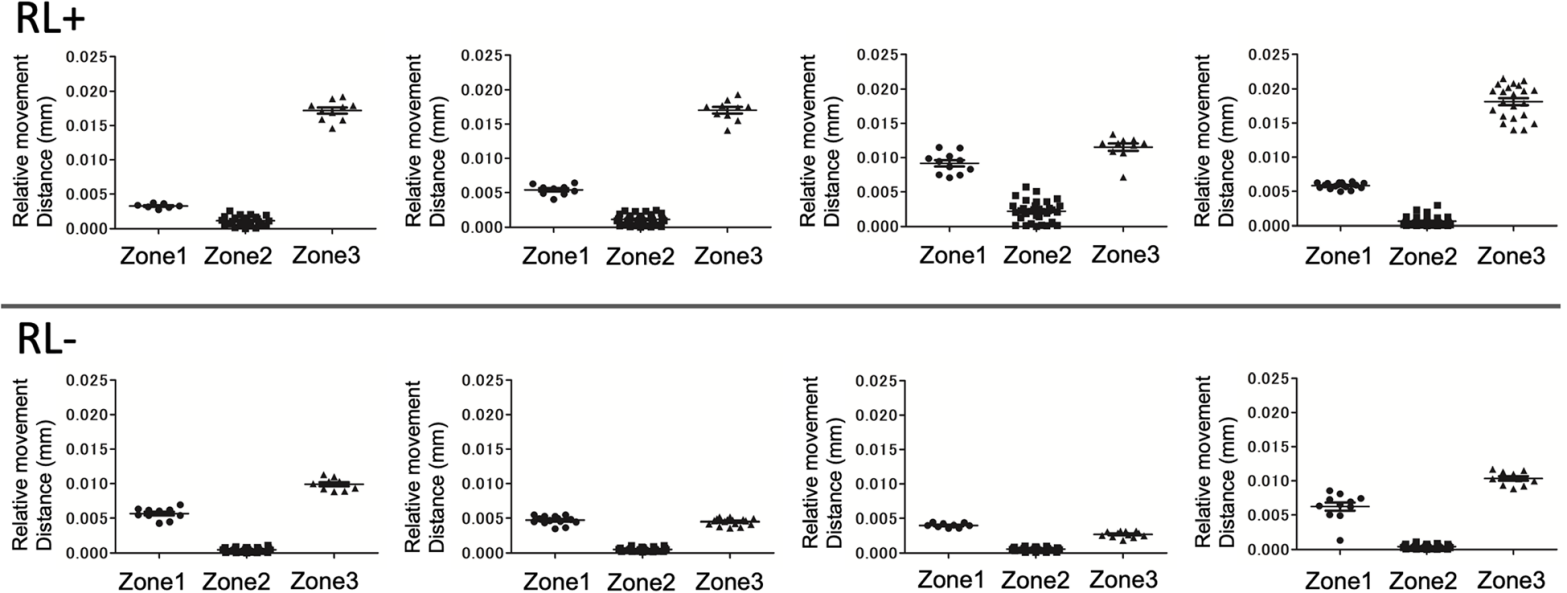
Element node relative movement distance in eight models with and without radiolucent lines (RLs)

In the study of micromotion, the relative movement distances of the element nodes were calculated to compare the three points corresponding to the zone of DeLee and Charnley (Fig. 4). The analysis was performed with eight models, four cases in which the radiolucent line appeared and four cases in which the radiolucent line did not appear, and 15 to 40 element nodes were extracted for each point (Fig. 5).

**Fig. 6:**
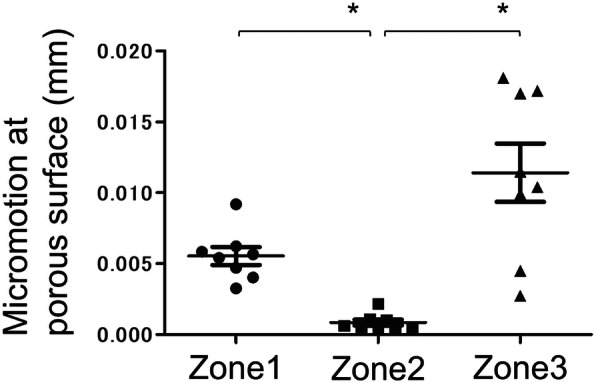
The micromotion at the porous region. Comparison of the SQRUM TT between the three groups. The micromotion was significantly larger at Zone 1 and Zone 3 than at Zone 2. *One-way analysis of variance, Dunn-Bonferroni test (*p* < 0.05)

By defining the mean of each point in each model as the micromotion, the micromotions of Zone 3 and Zone 1 were significantly greater than that of Zone 2. There was no significant difference between Zone 3 and Zone 1, but micromotion at Zone 3 tended to be large (Fig. 6).

**Fig. 7:**
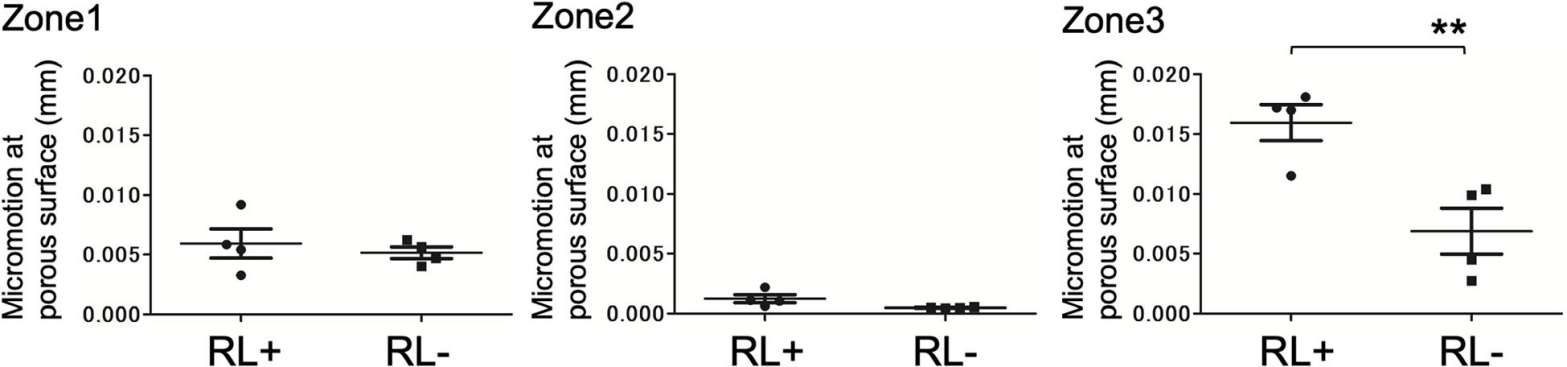
Difference in micromotion (mean [standard error]) for each point with and without radiolucent lines (RLs). There is no significant difference between Zone 1 (RL+: 0.005928 [0.001226], RL-: 0.005055 [0.0004913]) and Zone 2 (RL+: 0.00120 [0.0003337], RL-: 0.0004818 [0.00002564]), however, in Zone 3 (RL+: 0.01595 [0.001502], RL-: 0.006883 [0.001927]), the micromotion is significantly larger in the RL+ group than in the RL- group. ** Unpaired t-test (*p* < 0.05)

Furthermore, a comparison of cases with and without radiolucent lines revealed that micromotion was large in the cases in which radiolucent lines appeared at Zone 3 (Fig. 7).

## Discussion

The 3D highly porous metal cups are characterized by various features such as osteoinductive ability due to high porosity, reduction of stress shielding by elastic modulus close to cancellous bone, and high fracture strength of the porous region [21, 35–37]. It has been reported that the SQRUM TT, which was used in this study, can obtain biological immobilization from an early stage via the suppression of micromotion by scratch fit and physiological load transmission by the elastic modulus of the porous region similar to cancellous bone [10]. In our study, there were no cases of mid-term aseptic loosening, and no cases required reoperation for any reason.

Following the radiological examination, the initial gap disappeared during follow-up, but radiolucent lines were observed in 26 of 73 cases (35.6%). All cases were stable with a grade of II or higher according to the Modified DeLee and Charnley skeletal fixation score for the acetabulum. Imai et al. [16] also reported the presence of radiolucent lines, and it has been said that radiolucent lines appear in groups with less osteophyte formation.

In this study, the reason underlying the frequent appearance of radiolucent lines in DeLee and Charnley Zone 3 was analyzed using an FE model, focusing on micromotion during single leg standing. The micromotion at the point corresponding to DeLee and Charnley Zone 3 was significantly larger than that corresponding to Zone 2. Furthermore, the comparison of cases in which radiolucent lines did and did not appear indicated that micromotion was large in cases in which radiolucent lines appeared at the points corresponding to DeLee and Charnley Zone 3. These results suggest that the frequent appearance of radiolucent lines in DeLee and Charnley Zone 3 could be due to micromotion. Furthermore, SQRUM TT has a low modulus of elasticity in the porous region and close to the cancellous bone, which is designed to cause physiological load transmission and avoid stress shielding [21]. The result of the FE analysis focusing on the elastic modulus of the porous region suggested that the low elastic modulus may contribute to the generation of micromotion.

Porous factors, which determine the performance of a cementless acetabular component, are important and include porous design, surface processing, and metal species. A successful 3D porous structure requires not only the porosity and friction coefficient, but also the elastic modulus, which predicts micromotion. To date, surface treatments such as hydroxyapatite-coated and alkaline heat treatment have been applied to the porous area, and several authors have reported good clinical results [38–41]. As a material, a highly porous tantalum cup has been described to have good clinical results [7–9]. To achieve long-term results, further improvements in cementless cups are required, such as a combination of these elements.

When performing pressure load tests using cadaver bones, it is difficult to accurately evaluate the stress on the contact surface from the acetabular component implanted and the micromotion at the time of loading. Therefore, the FE analysis method presented here will aid the study of micromotion of the acetabular component, particularly focusing on the elastic modulus of the porous region.

The limitations of this study include the lack of implant survival rate evaluation over a long period as part of the clinical evaluation, limited number of FE analysis cases, and lack of implant size analysis. There was a significant difference in the cup inclination angle from immediately after surgery to the final observation, but the difference was minimal, and it is possible that postoperative changes in pelvic tilt had an effect. In addition, the analysis was performed only under the one-leg standing condition. In future studies, analysis under conditions such as walking and moving up and down stairs will provide more detailed results that reproduce more realistic phenomena.

## Conclusion

The mid-term clinical outcome of THA using a 3D porous titanium cup was excellent. An examination of micromotion by FE analysis showed that the emergence of radiolucent lines was predictable. Future improvements in implants with this research method can lead to longer-term clinical results.

## Data Availability

The datasets used and/or analyzed during the current study are available from the corresponding author on reasonable request.

## Abbreviations

THA: Total hip arthroplasty
3D: Three-dimensional
FE: Finite element
JOA: Japanese Orthopaedic Association
CT: Computed tomography
ROI: Region of interest
ICCs: Intraclass correlation coefficients
